# Critical Review of European Health-Economic Guidelines for the Health Technology Assessment of Medical Devices

**DOI:** 10.3389/fmed.2019.00278

**Published:** 2019-11-29

**Authors:** Maximilian Blüher, Sita J. Saunders, Virginie Mittard, Rafael Torrejon Torres, Jason A. Davis, Rhodri Saunders

**Affiliations:** Coreva Scientific, Königswinter, Germany

**Keywords:** medical device, health-technology assessment, guidelines, health economics, Europe, regulatory, systematic review

## Abstract

**Background:** Health-technology assessment (HTA) is a recognized mechanism to determine the relative benefits of innovative medical technologies. One aspect is their health-economic impact. While the process and methodology for pharmaceuticals is well-established, guidance for medical devices is sparse.

**Aim:** To provide an overview of the health-economic aspect in current European HTA guidelines concerning medical devices and identifying issues raised and potential improvements proposed in recent literature.

**Methodology:** Available guidelines by European agencies were each reviewed and summarized. To complement this, a full systematic review of current literature concerning potential improvements to existing HTA practices for medical devices, from PubMed and EMBASE, was conducted; the focus was on health economics. Authors could only review documents in English, French, or German. The systematic review yielded 518 unique articles concerning HTA for medical devices, 32 of which were considered for full-text review after screening of all abstracts.

**Results:** There is very limited consensus in—and mostly a complete lack of—guidance specific to medical devices in official HTA guidelines, for both clinical and economic analyses. Twenty two of 41 European countries had published official HTA guidance in English, French, or German. Among these only 4 (England, France, the Netherlands, and Sweden) dedicated a chapter or separate document to medical devices. In the literature, there is sufficient evidence to suggest medical devices need to be addressed separately from pharmaceuticals. However, mostly challenges are discussed rather than implementable solutions offered. We present the following set of frequently discussed issues and summarize any solutions that pertain to them: a weak evidence base, learning-curve effects, organizational impact, incremental innovation, diversity of devices, dynamic pricing, and transferability. We further combine reviewed information to suggest a set of possible best practices for health-economic assessment of medical devices.

**Conclusion:** For greater efficiency in medical-device innovation, European agencies should look to (re-)address the specific requirements of medical devices in their HTA guidelines. When both the health-economic and data requirements for the HTA of medical devices are defined, the development of practical solutions will likely follow.

## Introduction

The EU is moving toward a more unified health-technology assessment (HTA) process across member states (1). HTA involves the systematic evaluation of new healthcare interventions. It is a multidisciplinary process evaluating clinical (efficacy and safety) and non-clinical (economic, ethical, and organizational) aspects with the main purpose of informing policy decision making, e.g., reimbursement and pricing. National HTA agencies develop their own guidelines on general processes and methods for HTA; these are supplemented by guidance and best practice from international groups, such as the International Society for Pharmacoeconomics and Outcomes Research (ISPOR) and the European Network for Health Technology Assessment (EUnetHTA). Although the past has seen a greater emphasis on the clinical aspects of HTA, assessing economic value is growing in importance as healthcare budgets continue to account for an increasingly larger proportion of national gross domestic products^1^. The economics of healthcare provision is a local challenge, and it has been suggested that the outcomes and interpretation of economic evaluations help to explain the heterogeneity in coverage recommendations and decision-making across Europe (2). Indeed, how best to define value, quantify it, and compare it between health interventions is still an active debate (3). Still, the importance of health-economic analysis in HTA is unquestioned and growing. We focus here on reviewing all aspects required for a full health-economic analysis of medical devices.

Healthcare interventions are a diverse field of technologies, with pharmaceuticals accounting for the majority of healthcare expenditures (4). Likely, for this reason, guidelines for HTA were focused on pharmaceuticals. However, medical devices have garnered increasing attention in recent years. The European Parliament and Council published new regulations for medical devices in 2017 before which they had not been updated since the 1990s (5, 6). New regulations require compliance by May 2020, with major changes being an increased scope of devices (and software) under regulation and stricter rules for providing rigorous clinical evidence for higher-risk devices. Following this, more medical devices will need to be evaluated by HTA agencies than was previously necessary or even possible. Studies have highlighted that medical devices require a more flexible approach for HTA compared to pharmaceuticals and that guidelines often do not consider the intricacies of this rapidly evolving and highly heterogeneous field (7, 8). In particular, several differences exist between medical devices and pharmaceuticals with regard to health-economic analysis (7, 9–11), but there is little guidance on how garnered insights should be incorporated into the health-economic analysis or HTA process.

In this work, systematic reviews of (1) published HTA guidelines for Europe and (2) recommendations for medical-device economic evaluation were undertaken. The aim is to present the current European HTA landscape, gather and present recommendations for health-economic, medical-device assessment, and summarize these suggestions as a consolidated starting point of discussion on how the health-economic assessment of medical devices could be improved and formalized in policy.

## Methods

This review was conducted in two parts: (1) a review of current European, country-level guidelines for medical-device HTA and (2) a systematic review of current literature regarding recommendations specific to the development of guidelines for medical devices. Each guideline document in part 1 was reviewed by two authors, and part 2 followed a full systematic review as presented in *Preferred Reporting Items for Systematic Reviews and Meta-Analyses (PRISMA) recommendations* (12).

### Language

Only documents and publications in the three official European languages (English, French, and German) were considered.

### Search

#### Guidelines

The latest check for available guidelines was performed in April 2019.

#### Literature

The literature search was also performed in April 2019. Medical Subject Headings (MeSH) and text-word searches were used to identify literature of interest in PubMed and EMBASE. The structure of the PubMed search can be seen in Table 1, the corresponding EMBASE search framework is provided in the Supplementary Table 1. Only documents published between January 2000 and 31 December 2018 were selected for review. This cut-off date was taken to ensure that most eligible publications should have been fully indexed by April 2019.

**Table 1:** PubMed search performed in April, 2019.

**Index**	**Aim [category]**	**Search string**	**Hits**
1	Guidelines [MeSh]	guideline[mh]	153,099
2	Guidelines [Publication type]	guideline[pt]	32,427
3	Guidelines [Text Word]	guidelines[tw] OR guideline[tw] OR guidance[tw] OR guide[tw] OR “good practice”[tw] OR “good practice”[tw])	669,523
4	All guidelines	#1 OR #2 OR #3	672,412
5	Cost-effectiveness/benefit [MeSh]	Cost-Benefit Analysis[mh] OR Models, economic[mh]	86,487
6	Cost-effectiveness/benefit [Text Word]	“cost effectiveness”[tw] OR cost-effectiveness[tw] OR “cost benefit”[tw] OR cost-benefit[tw] OR “cost utility”[tw] OR cost-utility[tw] OR “cost outcome”[tw] OR cost-outcome[tw] OR “Health economics”[tw] OR “Health economic”[tw] OR “healthcare economics”[tw] OR “health care economics”[tw]	115,850
7	Medical device health economics	(#5 OR #6) AND (“medical devices”[tw] OR “medical device”[tw])	303
8	Health + Technology Assessment[Text Word]/[MeSh]	((health[tw] OR healthcare[tw] OR “health care”[tw]) AND (“technology assessment”[tw] OR “technology assessments”[tw] OR Technology Assessment, Biomedical[mh])) OR “medical device”[tw] OR “medical devices”[tw]	23,638
9	After 2000	2000/01:2018/12 [dp]	15,770,244
10	Guidelines OR Health + Technology Assessment	#4 OR #8	692,810
11	Final PubMed list	#7 AND #9 AND #10	92

**Final search string**: ((“Cost-Benefit Analysis”[mh] OR “Models, economic”[mh] OR “cost effectiveness”[tw] OR cost-effectiveness[tw] OR “cost benefit”[tw] OR cost-benefit[tw] OR “cost utility”[tw] OR “cost-utility”[tw] OR “cost outcome”[tw] OR cost-outcome[tw] OR “health economics”[tw] OR “health economic”[tw] OR “healthcare economics”[tw] OR “health care economics”[tw]) AND (“medical device”[tw] OR “medical devices”[tw])) AND ((guideline[mh] OR guideline[Publication Type] OR guidelines[tw] OR guideline[tw] OR guidance[tw] OR guide[tw] OR “good practice”[tw] OR “good practice”[tw]) OR ((health[tw] OR healthcare[tw] OR “health care”[tw]) AND (“technology assessment”[tw] OR “technology assessments”[tw] OR “Technology Assessment, Biomedical”[mh]))) AND 2000/01:2018/12 [dp].

### Information Sources

#### Guidelines

HTA guidelines for 41 European countries, listed in Supplementary Table 2, were downloaded through a manual search of their respective national/regional HTA or healthcare government-agency websites. If no guideline in one of the reviewed languages was identified, the authority was contacted via email to request whether any guideline was available in English, French or German.

#### Literature

Articles of interest were identified in the PubMed and EMBASE databases via structured, systematic searches.

### Study Selection

#### Guidelines

Only guidelines that referenced health economics, cost-effectiveness modeling, or the HTA process were selected. When multiple versions were available for a single country, the most recent one was reviewed. If guidelines were published in more than one of the reviewed languages, the English version took precedence. Where reference was made to previously-released guidelines, these were also reviewed. Guidelines from 22 European countries were identified for review. Included countries and guidelines are listed in Supplementary Tables 2, 3.

#### Literature

Returned articles from both PubMed and EMBASE were uploaded to *Sourcerer* (Covalence Research) for abstract screening. Duplicates were first removed and then at least two reviewers (MB, VM, RTT, and RS) independently screened the titles and abstracts against pre-defined exclusion criteria:

▪ not English, German, or French language
▪ formal irregularities (e.g., missing abstract)
▪ congress abstract/poster
▪ no economic considerations
▪ no HTA perspective
▪ not a recommendation for the improvement of HTA
▪ no medical-device focus
▪ narrow scope (e.g., product specific)

To determine whether criteria were being applied in a consistent manner, a 10% sample of the literature was screened, and results compared between reviewers. After establishing a clear and consistent understanding of exclusion parameters, the remaining literature was screened. Any discrepancies in screening results were resolved by discussion between reviewers and, if necessary, consulting a third reviewer. The quality and consistency of the systematic literature review were quantified with the Cohen's Kappa (κ) degree of agreement. Where a score of 0.61–0.80 is seen as substantial agreement, and above 0.81 is considered excellent agreement between reviewers.

Articles not excluded after title and abstract screening were obtained in full text and further evaluated against the exclusion criteria by two, independent reviewers. Those articles remaining after this stage were included for full analysis and any relevant data extracted (Supplementary Table 8).

### Data Extraction

Target data and characteristics for review and analysis (details below) were extracted from each document into a pre-specified, data-extraction form independently by multiple reviewers. MB reviewed all documents, while VM, JD, RTT, and RS each reviewed a portion of the results, ensuring that each of the documents was examined by at least two independent reviewers. Differences in extracted data were resolved through *post-hoc* discussion.

#### Guidelines

Several key pieces of information were defined to summarize guidelines (see extracted data in Supplementary Tables 4–7). These were informed by the recently published review of the implementation process of a Japanese HTA guideline by Shiroiwa et al., and included items such as perspective, type of analysis, discounting, and time horizon amongst others (13).

#### Literature

Any problems/issues associated with the HTA of medical devices and any potential solutions to them were extracted from the screened publications (Supplementary Table 8). After initial data extraction, the reviewers compared the collected data and determined a set of common topics. The problems and solutions were then indexed as relating to low-evidence, learning curve, organizational impact, incremental innovation, diversity, dynamic pricing, and/or transferability.

### Assessment of Quality and Bias

The assessment of risk of bias in the context of this systematic review is complex. There is no standard tool for assessment of risk of bias in guidelines—and by design, HTA guidelines represent the opinion and position of the health-policy framework that they form a part of. Similarly, the included peer-reviewed literature identified problems and made recommendations, which will inevitably be the opinion (however valid) of the authors. For this reason, risk of bias in these studies was assessed through identification of funding bodies and author disclosures. No formal scoring of these parameters was performed but the data are provided to give a perspective on the validity of the information provided by each study.

### Synthesis of Results

The extracted information was summarized and tabulated for review; and to identify and explore common themes. The most prevalent problems associated with the HTA of medical devices were compiled and the potential solutions summarized. Conflict, agreement, and synergy between recommendations were considered. Given the qualitative nature of the documents and data extracted, no statistical testing was performed.

## A Limited Consensus Exists Within HTA Guidelines of National Agencies

**Figure 1:**
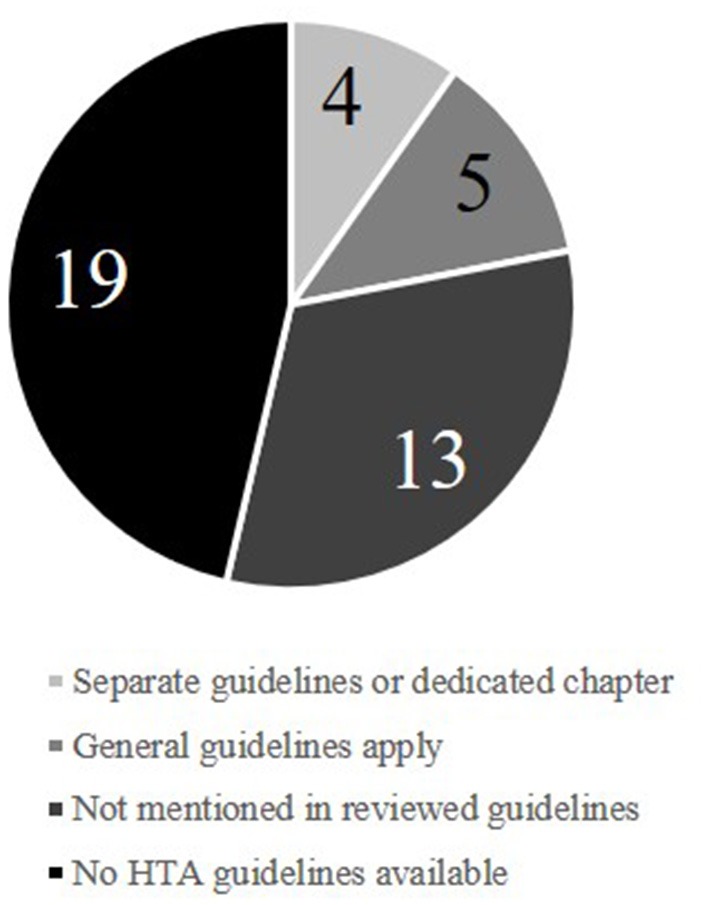
Medical-device representation in national HTA guidelines across Europe.

Amongst the 41 European countries (Supplementary Table 2) for which the availability of HTA guidelines was investigated, 22 (54%) had published official HTA guidelines in either English, French, or German (Figure 1). The identified guidelines were published between 1998 and 2017. The extracted recommendations for health-economic analyses are detailed in the Supplementary Material and summarized here.

Overall, the number of requirements for an HTA submission varied considerably between countries. England is an example with very specific guidance, including providing guidance on topics like equity or mapping that are rarely broached in other guidelines. English documentation even provides local pricing lists for medical interventions, as well as an HTA-application template. Other countries generally left more items to the applicant's judgment, providing only broad concepts to be interpreted and justified according to the needs of the product in question.

Guidelines mostly recommended a costing perspective of either societal or payer. Direct healthcare costs based on national prices were always to be used for any analysis. If a societal perspective was applied, then indirect costs were to be considered as well. Where a payer perspective was preferred, an additional analysis from the societal perspective was generally appreciated but not necessary. In six guidelines (Belgium, Croatia, England, France, Ireland, Scotland), the perspectives for outcomes and costs were separated. Cost effectiveness and cost utility were the preferred forms of analysis, while cost benefit and cost minimization were seen as potential additions. Only Austria, Belgium, and Russia mention budget-impact analysis as a complementary method. In all settings, the time horizon should be long enough to reflect all important differences in outcomes and costs. The only specific recommendations on the topic are at least 3 years for budget-impact analyses by Belgium and a preference for the expected patient lifetime by the Netherlands. The recommended yearly discount rate varied between three and five percent. Only two countries (Netherlands, Poland) differentiate between discounting for outcomes and costs. The discount rate is one of the prominent parameters of the sensitivity analysis, 12 guidelines state a separate rate (0–10%) for it. As guidelines generally recommended a cost-utility approach, quality-of-life measures were frequently discussed. Generic scores were preferred over disease-specific methods to derive quality of life. Among generic approaches, the EQ-5D questionnaire was mentioned most frequently.

In 13 of the 22 guidelines (59%), medical devices were not mentioned at all, while five (23%) stated that general guidelines apply for both pharmaceuticals and medical devices. Of the four specifying details for medical devices, three (England, France, and Sweden) provided medical-device–specific documents. In all three cases, most of the guidance provided focused on the clinical side of the HTA process and rarely provided specific requirements for economic analysis. The English guidance requires “*appropriate health-economic approaches to support decision-making”* (14). These approaches, however, are not specified beyond a 3.5% discount rate and inclusion of infrastructure, maintenance costs, and healthcare service outcomes (such as length of stay) (14). The medical-device–focused document for France deals mostly with the process of applying for reimbursement and choice of clinical study design (15). In the Swedish medical-device guidelines, the only detailed directive is the use of a societal perspective (16). The authors acknowledge the need for clear guidance as “*medical-device practices have a central and growing role in Swedish healthcare*” and note the HTA challenges that medical devices are facing (16). Though planned for the future, there is to date no recommended way forward to resolve the issues highlighted.

The Netherlands dedicated a chapter of their general guideline to medical-device–specific issues in HTA (17). This provided the most detailed information on the health-economic analysis of medical devices of any guideline considered here. In the Netherlands, a short-time horizon is suggested to account for the stepwise or incremental innovation common to medical devices. Further, intermediate endpoints such as ease of use, diagnostic performance, and duration of procedures should be considered. As the outcomes are not necessarily linked to quality-of-life changes for patients, a cost-effectiveness analysis is advised over a cost-utility analysis. The guidelines also specified that learning effects are to be regarded when extrapolating study results to real-life practice. Details on how to achieve this consideration were not included. Furthermore, discrete-choice experiment and multi-criteria decision analysis were proposed as alternatives to the EQ-5D when the value of a medical device is comprised of more than medical outcomes, such as the ease of use of a body-worn device.

## Issues Specific to Medical Devices are Mainly Unresolved in the Literature

**Figure 2:**
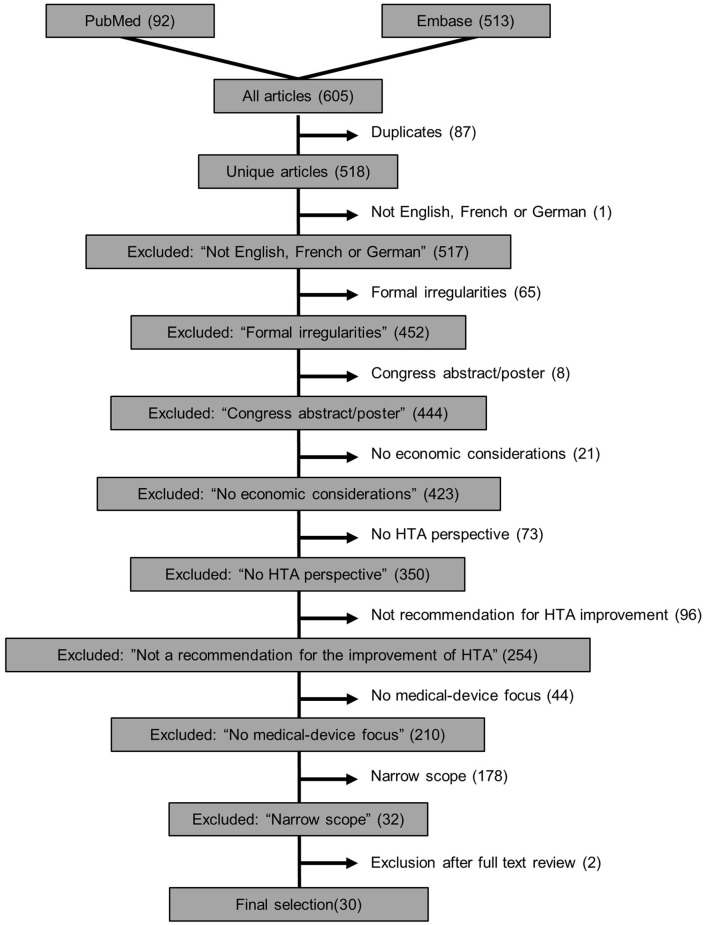
Flowchart of exclusion criteria in the systematic review of recommendations to improve HTA guidelines in relation to medical devices.

At least two authors working independently investigated each of the 518 articles included for abstract screening after duplicates were removed (Figure 2). An initial screening training set of 10% of the sample was assessed to ensure appropriateness of exclusion criteria and their consistent interpretation. The agreement in this training set was already substantial (κ = 0.64). After discussion of the discrepancies and which training articles to include/exclude, the remaining articles were screened. Across the entire set of documents, reviewer agreement was considerably higher (κ = 0.79). This demonstrated both the importance of the training set and the substantial agreement between independent reviewers. After screening of abstracts, 486 articles were removed according to the exclusion criteria and 32 publications were left for full-text review, of which another two were removed after full-text review (see Figure 2 and extracted data in Supplementary Table 8).

**Figure 3:**
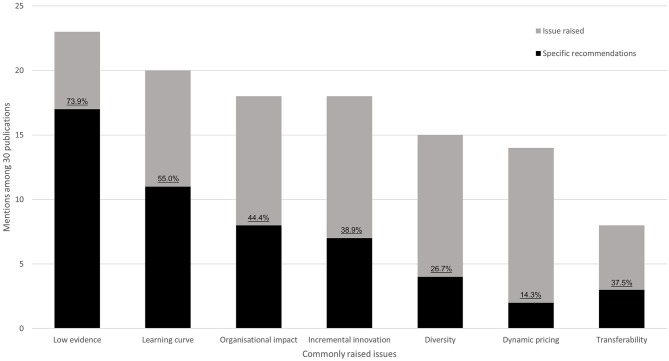
Key issues of medical device HTA identified in the literature. Stacked bars indicate the number of mentions of each issue. Black bars display how often solutions to these problems were proposed. Percentages indicate the ratio between solutions and mentions.

We identified seven common themes regarding unresolved issues and recommended solutions in the HTA of medical devices that were discussed in the literature: low evidence, learning-curve effects, organizational impact, incremental innovation, diversity, dynamic pricing, and transferability. In principle, each theme is important for health-economic assessment, however, several are general issues that are specific to medical devices as opposed to pharmaceuticals. Figure 3 plots the number of mentions for both issues and recommended solutions for an overview—and Supplementary Table 8 summarizes each publication separately. Three studies focused on a single topic (18–20), whereas the majority discussed multiple problems aiming to highlight the need for further research in the field of medical-device HTA and health-economic analysis (Supplementary Table 8). The most widely recognized issue was the low level of evidence that is currently available to demonstrate medical-device effectiveness (23 out of 30 reviewed publications—77%, Figure 3). Transferability is currently the least prominent issue (27%). In presenting a problem, authors did not always propose a potential solution but highlight a need for further research. Only for “low evidence” and “learning curve” did at least 50% of studies highlighting the issue also present a potential solution.

### Low Evidence

**Figure 4:**
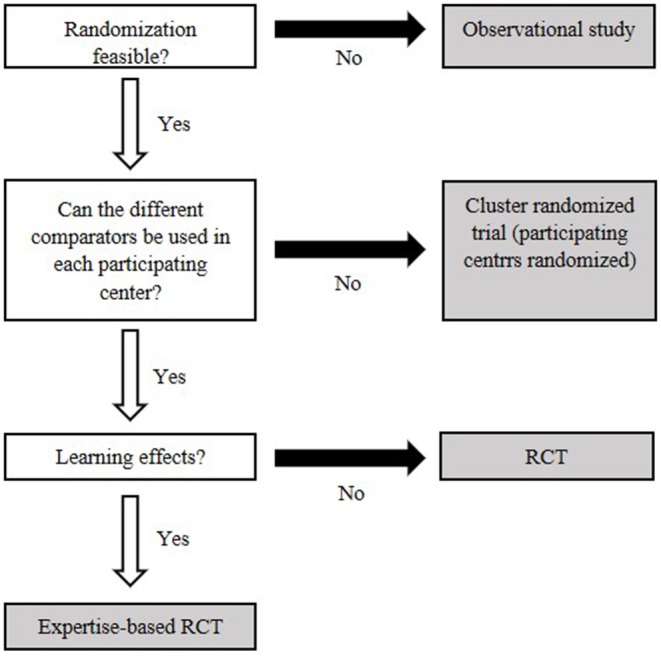
Decision flowchart to decide on an appropriate clinical study based on key features of the medical device. White boxes identify central characteristics. Gray fields represent types of clinical studies. Modified after Bernard et al. (22).

The most commonly mentioned issue was the lack of high-quality evidence for medical devices. This is likely linked to the difficulties of designing randomized controlled trials for medical devices, where blinding and proper randomization can be hard to implement (21). The most in-depth information provided for overcoming “low evidence” was provided by Haute Autorité de Santé (HAS), in their methodology guide on clinical evidence synthesis for medical devices (22). Here, suitable alternatives to pharmaceuticals' gold standard of randomized controlled trials are presented, and a decision tree is provided to facilitate making an appropriate selection (Figure 4) (22). Where new studies are not feasible, Bayesian methods were suggested for handling and synthesizing data from a wide variety of study designs. In total, 8 of the 30 publications indicated that use of Bayesian methods could be of benefit in the analysis of medical devices, though only one provided explicit examples or suggestions for use (8, 11, 20, 23–27).

Another option to minimize the impact of low-level evidence was suggested by Rothery et al., who highlight the potential of risk-sharing schemes for medical devices (25). Concepts such as “coverage with evidence development” allow early access to innovative technology while also incentivizing the generation of further evidence after market entry. This also shifts the HTA from a single-point decision to an adaptive process, which may help to address the issues of dynamic pricing and incremental innovation (28, 29). As such, the single HTA decision point would be replaced with a set of periodic (re)assessments to allow for an evolving real-world evidence base to be used to update guidance (28).

Current HTA guidance often refers to cost utility and quality of life (30). Many medical devices are, though, not therapeutic and so rarely impact patient quality of life directly; improved quality of care through increased acceptance or higher sensitivity or specificity is more likely (21). To account for this, Rosina et al. suggest using multiple-criteria decision analysis to measure outcomes to be utilized in cost-effectiveness analysis rather than comparing QALYs in a cost-utility analysis (21). It has also been suggested that a new questionnaire design could make cost-utility analysis feasible (31). Here, the theory was that the value of medical devices goes beyond direct clinical effects, impacting more on patient's sense of security, social interaction, sense of integrity, and convenience (31). Lesén et al. developed such a questionnaire to incorporate quality-of-life benefits beyond established clinical categories (31), and its use could supplement medical-device assessment and allow for a more meaningful cost-utility analysis. Comparison of this with cost-utility analyses from pharmaceuticals may, though, be inappropriate.

### Learning Curve Effects

Medical devices rarely have an impact on patient care without a healthcare professional being directly involved. Appropriate handling, interpretation, and intervention often require an initiation or training period to learn how to make best use of the medical device; medical outcomes can therefore display what is called a “learning curve” that reflects this period. In other words, full clinical benefits may be reached only after an initial introductory period due to potentially more complicated interventions as compared to pharmaceuticals. Bayesian methodologies were proposed for use in addressing the learning curve, though again, rarely was a specific solution provided (8, 20, 24, 32). Taking a real-world data approach, Varabyova et al. determined a generalizable model for estimating the learning curve (20). They suggested the collection of additional data during clinical trials to determine whether a learning effect for the intervention in question exists. To that end, appropriate endpoints, which might improve with experience, need to be defined beforehand. Examples of such endpoints could be in-hospital mortality or length of stay (20). Any procedure-specific learning effects identified should then be integrated into the developed model. Varabyova et al. suggested that this can be achieved through a Bayesian approach, with the collected data on the learning curve being used as informative priors (20).

### Organizational Impact

As opposed to pharmaceuticals, medical devices have a greater potential to indirectly impact an organization. Improvements, especially from an economic standpoint, can be gained by optimizing patient pathways or hospital workflows (26, 33, 34). Due to the learning curve, there may also be need for staff training, or the requirement for a sterilization process to be put in place for a reusable device.

### Incremental Innovation

Innovation in medical devices often happens in small but fast steps (11, 29, 35), examples being software upgrades or improved battery life. This poses the question whether and/or when it is necessary to reassess these small improvements, and if so, to which degree new assessments are needed.

### Diversity

Medical devices cover a wide variety of products, ranging from small, single-use disposables (e.g., syringes), to high-cost, long-term-use resources (e.g., magnetic resonance imaging machinery). This diversity creates a considerable hurdle when trying to establish standard procedures for assessment. Currently there is no solution for this problem; it is more a topic for debate (36, 37).

### Dynamic Pricing

Complex medical devices can often entail a high up-front investment with benefits only showing after longer periods of use (11, 18, 25, 35). Furthermore, list pricings, as commonly used for pharmaceuticals, generally do not exist for medical devices.

### Transferability

Organizational impact and learning curves increase the focus of medical-device HTA on local factors. This can make the transfer of assessment results between different settings more challenging than with pharmaceuticals (38, 39).

## Actionable recommendations

The recommended approaches toward overcoming the identified problems varied across publications. There was a general lack of consensus, with the number of potential solutions to each identified problem ranging from 2 to 17 (from 30 publications). Informed by our guideline and literature review, we suggest a framework that could be used as a stepping-stone toward development of best practice guidelines for medical device HTA. We focus on selecting the model parameters requiring assessment during a health-technology economic evaluation (see following subsections). The aim of the presented suggestions is primarily to increase debate on the topic and secondarily to provide some direction for medical-device HTA until official guidance is put in place. Key aspects to include are assessment of organizational impact, the learning curve, and impact of incremental innovation. Outside of a general lack of highest-level clinical evidence for efficacy and safety to support medical devices, these three issues were most commonly mentioned in the literature. While the following may not necessarily represent the order of priorities for every stakeholder, it is an indicator of issues for which multiple potential solutions exist.

### Medical-Device Impact

Each medical device interacts with either a patient and/or a provider. Determine whether the patient/provider interaction is transient (≤1 month), short term (>1 month and ≤1-year), or long term (>1 year). The impact of interaction length can influence recommendations made for perspective, timeline, and type of economic analysis to implement (see below) and perhaps other issues as well.

### Categorization

To simplify the problems of diversity, stratification can be useful. Here, we recommend to: (1) determine whether the device in question is therapeutic, diagnostic, monitoring, or other; (2) differentiate between patient-used, implanted, and assistive for medical personnel; and (3) identify the risk level of the device in the setting in question (36). These categories help the researcher to understand the relevant endpoints and, therefore, suitable types of studies and analysis.

### Clinical Evidence

Early planning for clinical and economic data is critical and the French Haute Autorité de Santé publication is recommended as a starting point for the decision process (22). An economic analysis can only use the evidence available, but where multiple sources exist, the highest level of evidence should be used or evidence synthesis undertaken. If local data exist, a scenario analysis replacing the highest level of evidence with these local data should be undertaken.

### Perspective

The perspective can often be adopted from the country's general guidelines. If none is specified, we recommend the hospital's or payer's perspective for medical devices with transient or short-term patient/provider interaction. As long-term interactions could have substantial societal impact, this perspective should also be taken in countries accepting this approach, with results stated separately for the payer and societal perspective.

### Method of Analysis

When specified, guidelines often include cost-utility analysis. However, quality-of-life measures can be problematic for transient and short-term medical devices (31). Cost-effectiveness and/or budget-impact analysis for transient and short-term medical devices is likely more appropriate. Medical devices with longer-term interaction could use cost-utility analysis.

### Organizational Impact

When introducing a medical device into a healthcare setting, it can change—or could benefit from changes in—the way in which health services operate: for example, changes in care pathways or staffing requirements, equipment procurement and cleaning processes, or in staff–patient interactions. If this is the case, a thorough assessment of the organizational impact must be made. An organizational impact assessment may require a discrete-event simulation to estimate or quantify the impact a pathway change will have on the patient/provider level. Any changes will also likely require staff training/education.

### Learning Curve

In general, include the potential to have a learning curve for any model developed for HTA of medical devices. As data availability to inform on specific learning curves is rare, the learning curve should have inbuilt flexibility. For example, model the learning curve with parameters included for time or number of procedures before maximal impact (20). Consider staff or patient training and education, and associated time and cost factors.

### Time Horizon

For transient medical devices, a time horizon of maximally 3 years is likely sufficient. Short-term devices should use up to a 5-year time horizon. For long-term devices, a time horizon that extends 5 years beyond the expected patient/provider interaction is likely sufficient. For diagnostic devices, the time horizon should extend shortly past the point of scheduled retesting or the time at which disease impact would be expected. Given the incremental nature of medical-device development, a time horizon of longer than 20 years is not recommended. These suggestions are rough estimates, each medical device is different. Therefore, the optimal time horizon must be decided on considering all intricacies of the device in question and the availability of follow-up data.

### Discount Rate

The use of country-specific discount rates as stated in most general guidelines is recommended. In the absence of guidance, EUnetHTA recommends the use of 3–5% as do most recommendations in European guidelines (see Supplementary Table 5). In the sensitivity analysis, a broader range around this value should be explored, of 0–10%. Unless otherwise specified, apply the same discount rate to costs and utilities.

### Method to Derive Quality-of-Life Score

When there is direct patient contact with the medical device, consider using the MedTech20 questionnaire^2^. This generic questionnaire was specifically designed for assessing medical-device impact on quality of life and is currently available in English, Swedish, and Norwegian (31). For general health, EQ-5D and SF-6D are wide-spread measures that can be used in economic analyses (40); most national HTA guidelines give EQ-5D as the preferred measure (Supplementary Table 5) and it should be used if no local guidance is provided.

### Costs to Be Included

Local costs from the payer perspective should always be included. Indirect costs, such as carers and time off work, can be included if the societal perspective is analyzed. Which costs need to be included is generally decided by each country. As this decision is generally not dependent on what kind of product is investigated, guidance can usually be found in each country's general HTA guideline.

### Incremental Innovation and Dynamic Pricing

It is almost impossible to account for incremental innovation and dynamic pricing in a single health-economic model. An assessment beginning early in product development and lasting beyond initial market entry may help inform expectations of future improvements and changes in pricing. This, though, increases the length of time required for the HTA. Early decisions and programs such as “coverage with evidence development,” can be beneficial for all stakeholders in such a prolonged process (25). Another option is to consider risk-sharing agreements between the medical-device company and payer, such that introducing new devices comes at minimal financial risk and payment is linked to improved patient care or hospital efficiencies.

### Sensitivity Analysis

Some commonly needed additional parameters for the health-economic assessment of medical devices are presented here, though not all of these apply to all products:

▪ Learning curve effects (20)
▪ Potential future price and outcome change due to incremental innovation
▪ Organizational impact

## Discussion

Almost half (19 of 41) of European countries do not provide guidance for HTA health-economic assessments (Figure 1). On the one hand, the reasoning for establishing an HTA processes is plain: limited resources in the face of increasing healthcare costs make a rational decision process based on both clinical and economic evidence highly advantageous. On the other hand, a lack of guidance on the process complicates it considerably: it can become harder for manufacturers to produce suitable HTA reports while also complicating the evaluation of submissions for regulators, as there are no standards for comparison. A lack of clear HTA guidance can thus waste resources and increase the risk of non-optimal decisions. Acknowledging the value of HTA, the European Commission promoted the establishment of a European HTA network^3^. In 2005, led by the Danish Center for HTA (DACEHTA), the EUnetHTA project was launched to strengthen communication and cooperation between European HTA agencies. To this day, the EUnetHTA helps to coordinate joint research efforts and provides information to support the establishment of new or updated HTA processes.

In addition to missing guidance for general HTA, only 10% (4 of 41) of the investigated countries provide any medical-device–specific information. Despite the literature showing that medical devices differ considerably from pharmaceuticals with respect to health-economic assessment (7, 10), it is clear that even among countries with established HTA guidelines, medical-device–specific instructions are rare. When information on medical devices was provided, it was sparse and focused on clinical evidence. While clinical outcomes are an important aspect of HTA, only a comprehensive consideration of all aspects relevant to medical-device assessment can fulfill the purpose of HTA. Economic assessment is an important part of this, and its relevance is only expected to increase as healthcare budgets come under further pressure from aging populations and increasing innovation.

In contrast to the limited information available in national guidelines, interest in medical-device assessment is increasing on a European level, which is shown by the MedtecHTA project,^4^ funded under the European Commission's 7th Framework Programme. This 3-year, multi-national project began with the evaluation of the current state of practice in medical-device HTA methodology in 2013 and finished with recommendations on how to improve the existing processes in 2017. With MedtecHTA collaborating with EUnetHTA, EUnetHTA members may become more aware of medical-device–specific issues. This collaboration might be an opportunity to strengthen the cooperation between European states to increase consistency of medical-device requirements across them. Standardization has the potential to speed up availability of innovative medical devices, not just in individual countries but across Europe. As of 2018, work toward standardizing HTA in the EU is underway, as shown by the recently released proposal for an EU-wide regulation of HTA (1). Although decisions on reimbursement and pricing and establishing criteria for such decisions remains a national concern, i.e., European-wide regulations on economic assessments are prohibited by law (1), current awareness of the need for change could provide an opportunity to develop best-pratice guidance and a support framework for economic analyses of medical devices. General guidelines proposed by the EU could then be adapted to reflect the needs of individual countries and ideally be published by their own HTA agencies. The increased speed of uptake of innovative technology would be facilitated by the possibility to transfer parts of the HTA performed in one country to another: e.g., the proposed regulation requires member states to take full account of results of a joint clinical assessment and to not repeat these (1). While standardization is enticing, there is a fine balance between generalization leading to better transferability and oversimplification leading to the omission of localized practices or situations—and, therefore, inaccurate predictions of real-life results.

An inescapable conclusion from the review of current literature is that problems with medical-device, health-economic assessment are known (7, 10), however, solutions are few (Figure 3). Still, certain areas of medical-device evaluation are suitably established and informed to warrant guidance on aspects to be, at a minimum, considered during a health-economic assessment. With this review, we provide some actionable items including the following: (1) stratify medical devices to simplify the selection of model parameters; (2) provide the option to model a learning curve, i.e., the time taken, or number of procedures required, to achieve the maximal benefit when introducing a new medical device (20); (3) perform an organizational-impact assessment of how the introduction of a new device changes the way in which healthcare services operate; and (4) consider staff and/or patient training and education requirements and costs associated with both learning-curve effects and organizational impact.

Insufficient, high-quality clinical evidence to support medical device efficacy and safety was the main point of discussion in the literature (Figure 3). Clinical evidence is not only required for establishing efficacy and safety but comparison of clinical outcomes with the standard of care is a basic requirement for several economic analyses, such as a cost-effectiveness analysis. With the new medical-device regulations [Regulation (EU) 2017/745-746], published in 2017 with compliance required from May 2020, the greater demand for clinical studies should increase available data on clinical outcomes in the future, especially for medical devices categorized into higher-risk classes (5, 6). A greater volume of clinical data will enable agencies to fully assess more medical devices than was previously possible; and more published reports should naturally increase the focus of HTA agencies on issues specific to medical devices.

Beyond potential improvements to health-economic modeling, bringing beneficial medical devices to patients and providers in the shortest possible time is of importance. Here there are real-world examples of how risk-sharing agreements and “coverage with evidence development” can help to balance timely access to innovative technology while generating continued evidence of clinical and economic effectiveness (25). Although the nature and advantage of risk-sharing agreements is much discussed, interest in such agreements for healthcare provision has steadily increased in recent years (41).

At this time, most of the research is focused on identifying gaps and inconsistencies concerning issues with the HTA assessment of medical devices. Only with clear guidance on how authorities wish these issues to be overcome, or on the outcomes/answers that they wish to see, is it likely that we will see an increase in methods and solutions to move medical-device economic assessment forwards.

## Conclusion

Medical devices play an important role in healthcare provision, yet the HTA guidelines for their assessment are lacking. There is sufficient evidence to support the development of HTA processes and methodologies for medical devices that are clearly separate to those for pharmaceuticals; and thus, HTA guidelines must reflect these differences. The literature presents several issues, however, practical solutions that are shown to improve economic analyses of medical devices still need to be developed. Accounting for the learning curve, organizational impact, and dynamic pricing is important, but little guidance is available on how to achieve this. We recommend carefully considering the medical-device patient or physician interaction length to inform the type of model best suited to its health-economic analysis. Most urgently, we call on relevant authorities to determine how they wish to move forward, thus allowing research to start answering many of the currently open questions.

## Author Contributions

RS conceived the idea. MB and RS searched for literature and guidelines. MB, RS, and VM performed the literature screening. MB, RS, VM, JD, and RT extracted data. MB, RS, SS, VM, and RT collated and summarized the data into the presented work. MB and SS wrote the manuscript, which was edited by RS and SS. All authors reviewed and approved the manuscript.

### Conflict of Interest

All authors work at Coreva Scientific GmbH & Co. KG, which is a health-economics and value-based healthcare consultancy with a focus on medical devices.

## Footnotes

^1^https://www.theglobaleconomy.com/rankings/Health_spending_as_percent_of_GDP (accessed September 10, 2019).

^2^MedTech20 official website — http://www.medtech20.com/

^3^https://www.eunethta.eu/ (accessed September 10, 2019).

^4^www.medtechta.eu (accessed September 10, 2019).
