# Supplementary material for: Critical Review of European Health-Economic Guidelines for the Health Technology Assessment of Medical Devices
Source: Front Med (Lausanne). 2019 Nov 29;6:278. doi: 10.3389/fmed.2019.00278 (PMC6895571; doi:10.3389/fmed.2019.00278)
Supplement: Supplementary file 1 [file Data_Sheet_1.pdf]

Supplementary Material to

Critical Review of European Health-Economic Guidelines for the Health Technology Assessment of Medical Devices

by Maximilian Blüher et al.

Contents

1 Additional search strings used in the systematic literature review 2
2 Availability of HTA guidelines in European countries 3
3 National HTA guidelines 6
3.1 Perspectives and type of analysis for economic models 8
3.2 Economic modelling specifics: time horizon, discount rate, quality of life 10
3.3 Costs to be included in economic models 12
3.4 Guidelines specific to medical devices 14
4 Key issues extracted from the literature 15
5 Acronyms used in the supplementary tables 22
6 References 23

The supplementary data is comprised purely of tables, however, to provide some overview and ease of navigation, they have been divided into sections.

1 Additional search strings used in the systematic literature review

Supplementary Table 1: Additional search strings used for the systematic literature review.

Category	Search string
**Embase text**	('cost effectiveness analysis':ab,ti,de OR 'cost utility analysis':ab,ti,de OR 'cost benefit analysis':ab,ti,de OR 'cost minimization analysis':ab,ti,de OR 'cost outcome':ab,ti,de OR 'health care economics':ab,ti,de OR 'health care cost':ab,ti,de OR 'pharmacoeconomics':ab,ti,de) AND 'medical device*':ab,ti,de AND (('health technology assessment':ab,ti,de OR 'biomedical technology assessment':ab,ti,de) OR ('guideline*':ab,ti,de OR 'good practi*e':ab,ti,de)) AND (([english]/lim OR [french]/lim OR [german]/lim) AND [2000-2018]/py)
**Emtree keyword**	('cost effectiveness analysis'/exp OR 'budget impact analysis'/exp OR 'health economics'/exp) AND 'medical device'/exp AND 'biomedical technology assessment'/mj AND [2000-2017]/py AND ([english]/lim OR [french]/lim OR [german]/lim)

2 Availability of HTA guidelines in European countries

Supplementary Table 2: HTA guideline availability across 41 European countries in alphabetical order ('/' indicates that no data was identified).

Country	HTA guideline	Medical devices
Albania	Email sent, no answer received	/
Austria	Methodenhandbuch für Health Technology Assessment Version 1; 2012[1]	General guidelines apply
Belgium	Belgian Guidelines for Economic Evaluations and Budget Impact Analyses: Second Edition; 2012[2]	General guidelines apply; clear regulation of MDs planned
Bosnia and Herzegovina	Email contact, No guidelines in place	/
Bulgaria	Methodological Recommendations for presented documentation for assessment of the efficacy, safety, and pharmacoeconomic parameters of medicinal products applying for inclusion in the Positive Drug List; 2015[3]	Not mentioned
Croatia	The Croatian Guideline for Health Technology Assessment Process and Reporting; 2011[4]	General guidelines apply
Cyprus	Email contact, No published guidelines for HTA	/
Czech Republic	Email sent, no answer received	/
Denmark	Health Technology Assessment Handbook Copenhagen: Danish Centre for Health Technology Assessment, National Board of Health; 2007[5]	Not mentioned
England	Guide to the Methods of Technology Appraisal, 2013[6]	Separate guideline[7]
Estonia	Baltic Guideline for Economic Evaluation of Pharmaceuticals (Pharmacoeconomic Analysis); 2002[8]	Not mentioned
Finland	Preparing A Health Economic Evaluation to be Attached to the Application for Reimbursement Status and Wholesale Price for a Medicinal Product; 2015[9]	Not mentioned (additional guideline available in Finnish might include MDs)
France	Choices in Methods for Economic Evaluation; 2012[10]	Separate guideline[11]

Germany	IQWiG Methoden 5.0; 2017[12]	Not mentioned
Greece	No suitable contact address could be identified	/
Hungary	Professional Healthcare Guideline on the Methodology of Health Technology assessment; 2017[13]	Not mentioned
Iceland	Email sent, no answer received	/
Ireland	Guidelines for the Economic Evaluation of Health Technologies in Ireland; 2014[14]	General guidelines apply
Italy	Email sent, no answer received	/
Luxembourg	Email sent, no answer received	/
Latvia	Baltic Guideline for Economic Evaluation of Pharmaceuticals (Pharmacoeconomic Analysis); 2002[8]	Not mentioned
Lithuania	Baltic Guideline for Economic Evaluation of Pharmaceuticals (Pharmacoeconomic Analysis); 2002[8]	Not mentioned
Macedonia	Email sent, no answer received	/
Malta	Email sent, no answer received	/
Moldova	Email sent, no answer received	/
Montenegro	Email sent, no answer received	/
Netherlands	Guideline for Economic Evaluations in Healthcare; 2016[15]	Dedicated chapter
Norway	Guidelines on how to Conduct Pharmacoeconomic Analyses; 2012[16]	Not mentioned (guideline only available in Norwegian might address MDs)
Poland	Health Technology Assessment Guidelines Version 3.0; 2016[17]	General guidelines apply
Portugal	Guidelines for Economic Drug Evaluation Studies; 1998[18]	Not mentioned
Romania	No suitable contact address could be identified	/
Russia	Procedure for Clinical and Economic Evaluation of Drug Lists that are Submitted for Reimbursement Coverage from Public Health Care Budget; 2010[19]	Not mentioned
Wales	Generally follows NICE guidance	/
Scotland	Guidance to Manufacturers for Completion of New Product Assessment Form (NPAF); 2017[20]	Not mentioned

Serbia	Email sent, no answer received	/
Slovakia	Email sent, no answer received	/
Slovenia	Email sent, no answer received	/
Spain	Spanish Recommendations on Economic Evaluation of Health Technologies; 2010[21]	Not mentioned
Sweden	Assessment of Methods in Health Care: A Handbook (2017)[22] & General Guidelines for Economic Evaluations from the Pharmaceutical Benefits Board (2013)[23]	Separate guideline[24]
Switzerland	Email contact, currently EUnetHTA guidelines are used. Swiss-specific guidelines are in development.	/
Ukraine	Guidelines in development	/

3 National HTA guidelines

Supplementary Table 3: List of national HTA guidelines and date of publication.

Country	Source	Year
Austria	Methodenhandbuch für Health Technology Assessment Version 1	2012
Belgium	Belgian Guidelines for Economic Evaluations and Budget Impact Analyses: Second Edition	2012
Croatia	The Croatian Guideline for Health Technology Assessment Process and Reporting	2011
Denmark	Health Technology Assessment Handbook Copenhagen: Danish Centre for Health Technology Assessment, National Board of Health	2007
England	Guide to the Methods of Technology Appraisal	2013
Europe (EUnetHTA advice, not binding)	Methods for Health Economic Evaluations - A Guideline based on Current Practices in Europe	2015
Estonia, Latvia, Lithuania	Baltic Guideline for Economic Evaluation of Pharmaceuticals (Pharmacoeconomic Analysis)	2002
Finland	Preparing A Health Economic Evaluation to be Attached to the Application for Reimbursement Status and Wholesale Price for a Medicinal Product	2015
France	Choices in Methods for Economic Evaluation	2012
Germany	IQWiG Methoden 5.0	2017
Hungary	Professional Healthcare Guideline on the Methodology of Health Technology assessment	2017
Ireland	Guidelines for the Economic Evaluation of Health Technologies in Ireland	2014
Netherlands	Guideline for Economic Evaluations in Healthcare	2016
Norway	Guidelines on how to Conduct Pharmacoeconomic Analyses	2012
Poland	Health Technology Assessment Guidelines Version 3.0	2016
Portugal	Guidelines for Economic Drug Evaluation Studies	1998

Russia	Procedure for Clinical and Economic Evaluation of Drug Lists that are Submitted for Reimbursement Coverage from Public Health Care Budget	2010
Scotland	Guidance to Manufacturers for Completion of New Product Assessment Form (NPAF)	2017
Spain	Spanish Recommendations on Economic Evaluation of Health Technologies	2010
Sweden	Assessment of Methods in Health Care: A Handbook & General Guidelines for Economic Evaluations from the Pharmaceutical Benefits Board	2017 & 2003

3.1 Perspectives and type of analysis for economic models

Supplementary Table 4: Perspectives and method of economic analysis specified in national HTA guidelines.

Country	Perspective	Method of analysis
Austria	Depends on research question	CEA, CUA, (BIA as support)
Belgium	Costs: Health care payers (federal government, communities & patients)<br>Outcomes: Society	CEA or CUA; choice should be justified<br>BIA as complementary
Croatia	Costs: public payer (societal perspective can be added)<br>Outcomes: All health effects on individuals	CEA or CUA
Denmark	Societal perspective	CMA, CEA, CUA or CBA
England	Outcomes: All direct health effects, whether for patients or, when relevant, carers Costs: NHS and PSS	CUA with fully incremental analysis
Europe (EUnetHTA advice, not binding)	Health care or Societal perspective	CEA and CUA
Estonia, Latvia, Lithuania	Health care perspective (societal can be performed additionally)	CEA and CMA or CUA
Finland	Not specified (included costs indicates payer's perspective)	CMA, CEA, CUA (preferred), or CBA
France	Collective perspective on cost: All health care funders<br>On health effects: Population whose health is affected and the general public	CUA and/or CEA
Germany	Insurer, societal, social care. specifics vary based on the research question	CEA preferred, CUA only for very specific populations
Hungary	Payer perspective is recommended<br>Besides that, if possible, societal perspective	CUA, complemented with CEA

Ireland	Benefits: Individuals<br>Costs: Publicly-funded health & social care system	CUA
Netherlands	Societal perspective	CUA
Norway	Societal perspective (with some limitations: exclude consideration of added life years and tax funding)	CUA preferred, CVA possible supplement, CMA in certain circumstances
Poland	Public payer's perspective and joint perspective of the public payer and the beneficiaries	CUA, CEA, CMA or CCA (CBA not recommended, and choice must be justified)
Portugal	Societal perspective	CUA (preferred), CMA, CEA, CBA
Russia	Perspective of the society as a whole, or the<br>public health system	CMA, CEA, CBA, CUA. Disease cost analysis and BIA can be considered in addition
Scotland	Outcomes: all direct health effects whether for patients or, where relevant, other individuals<br>Costs: NHS in Scotland and social work	CUA
Spain	Societal (in addition third-party NHS)	CMA, CEA, CBA, CUA
Sweden	Societal perspective	CUA recommended (other analyses possible if justified)

3.2 Economic modelling specifics: time horizon, discount rate, quality of life

Supplementary Table 5: Time horizon, discount rate, and method to derive QOL score extracted from national HTA guidelines.

Country	Time horizon	Discount rate	Method to derive QOL score
Austria	Long enough to reflect all important differences in costs or outcomes	3% (0,5 & 10% for sensitivity analysis)	Standardized indirect methods (EQ-5D etc.)
Belgium	CEA/CUA: long enough to reflect all costs and outcomes<br>BIA: Min 3 years, simulate until a steady state of budget-impact is reached	CEA/CUA: 3% on costs and 1.5% on outcomes. BIM: No discounting	Generic QOL instrument such as the EQ-5D
Croatia	Long enough to reflect all important differences in costs or outcomes	5% on both costs and health effects (sensitivity analyses between 3% and 10%)	EQ-5D is the preferred measure
Denmark	Not specified	Not specified	Generic and disease-specific instruments separately
England	Long enough to reflect all important differences in costs or outcomes	3.5%	The EQ-5D is the preferred measure
Europe (EUnetHTA advice, not binding)	Long enough to reflect all important differences in costs or outcomes	3-5% (variety of rates should be explored in sensitivity analysis)	EQ-5D recommended (HUI, SF-6D or 15D also acceptable), based on the general public

Estonia, Latvia, Lithuania	Model a sufficient analysis period when trial data provide too short a time frame	5%	EuroQol and HUI recommended
Finland	Time horizons can be e.g. one, five and ten years, as well as a period of time corresponding to the length of the clinical trial that the evaluation is based on.	3%	Validated generic QoL measure
France	Long enough to reflect all important differences in costs or outcomes	4%, after 30 years, the discount rate linearly declines to 2%. (3-6% in sensitivity analysis)	EQ-5D & HUI 3 are preferred (based on public preferences)
Germany	Long enough to reflect all important differences in costs or outcomes	3% (0,5% in sensitivity analysis)	Generic and specific methods (use of QALYs is generally not recommended)
Hungary	Long enough to reflect all important differences in costs or outcomes & should also be adjusted to the life expectancy of the Hungarian population, taking the disease-specific mortality into account	3.7% (costs: 2-5%; health effects: 0-5% in sensitivity analysis)	EQ-5D, SF-36 or mapping of disease-specific questionnaires
Ireland	Long enough to reflect all important differences in costs or outcomes	5% (0-6% for sensitivity analysis)	EQ-5D or SF-6D
Netherlands	The time horizon required for an economic evaluation should preferably cover the expected lifetime (different time horizon may be chosen, however, if well-argued)	Costs 4%; effects 1.5%	EQ-5D validated for Dutch context (life year, when relevant)
Norway	Long enough to reflect all important differences in costs or outcomes	4%	Multi-attribute utility (MAU)-instruments (e.g. EQ-5D, SF-6D and 15D)
Poland	Long enough to reflect all important differences in costs or outcomes (length	5% costs, 3.5% outcomes	EQ-5D preferred

	of the time horizon should be tested as part of a sensitivity analysis)	(0% for both in sensitivity analysis)	
Portugal	Long enough to reflect all important differences in costs or outcomes	5% (also for sensitivity analysis)	Any value-based method validated for Portugal
Russia	Not specified	5%	Method must be specified
Scotland	Long enough to reflect all important differences in costs or outcomes	3.5% (0% & 6% in sensitivity analysis)	Validated generic utility instrument such as the EQ- 5D preferred
Spain	Long enough to reflect all important differences in costs or outcomes	3% (0-5% for sensitivity analysis)	Disease-specific, validated
Sweden	Long enough to reflect all important differences in costs or outcomes	3% (0 & 5% for sensitivity analysis)	EQ-5D, SF-6D, HUI-3 or standard gamble / time trade-off

3.3 Costs to be included in economic models

Supplementary Table 6. Costs to be included as specified by national HTA guidelines.

Country	Costs to be included
Austria	Depends on the chosen perspective
Belgium	CEA/CUA: Health care costs paid out of the health care budget, by the federal government, the communities and the patients.<br>BIA: Treatment effects, side effects, and other short- or long-term consequences
Croatia	Direct cost relevant to Croatian Institute for Health Insurance (where measurable and relevant, indirect costs and cost falling outside of the Croatian Institute for Health Insurance should be reported separately)
Denmark	Direct & indirect

England	Costs should relate to NHS and PSS resources and should be valued using the prices relevant to the NHS and PSS
Europe (EUetHTA advice, not binding)	All direct health care costs should be included in the main analysis (recommended to present costs borne by other sectors of the society, e.g. indirect costs in an additional analysis when relevant)
Estonia, Latvia, Lithuania	All direct costs (for societal perspective: both direct and indirect costs)
Finland	All direct health care and comparable social welfare costs
France	All the resources consumed in the production of the intervention, whatever the source of funding (direct costs). Indirect costs can be analysed separately
Germany	Depends on the specific perspective chosen (table on page 94)
Hungary	Payer perspective: direct costs<br>Societal perspective: all benefits and costs (direct and indirect costs within and outside of the healthcare system)
Ireland	Only direct costs relevant to the publicly-funded health and social care system
Netherlands	All costs inside the healthcare sector, patient and family and other sectors.
Norway	Drug costs, hospital costs, care costs and any costs associated with the production effects
Poland	Direct medical costs, direct non-medical costs and indirect costs. Details provided
Portugal	Direct costs of providing health care, the costs of social services and other sectors related to health care and the costs borne by patients and their families
Russia	Position of society: all costs associated with the use of drugs are taken into account<br>Public health system: only costs of the public health system of the appropriate level are taken into account
Scotland	Costs should relate to resources that are under the control of the NHS in Scotland and social work
Spain	Separate productivity losses, opportunity costs of resources, health care relevant costs
Sweden	All relevant costs and revenues for treatment and ill health, irrespective of the payer.

3.4 Guidelines specific to medical devices

Supplementary Table 7: Medical-device specific guidelines in national HTA guidelines.

Country	Medical devices
Austria	Guidelines apply to medical devices but there are no specific regulations
Belgium	In general, the recommendations from this report should be integrated in the legislation about the reimbursement of drugs, medical devices and medical interventions. products, data requirements and data availability for medical devices lag behind. So far, there is no requirement to perform an economic evaluation nor an assessment of the budgetary impact of new devices for which reimbursement is requested. Even if this is not an obligation (yet), economic evaluations are nonetheless useful for medical devices and other health care interventions in order to stimulate the efficient use of our limited resources. Therefore, these guidelines are also useful for devices and other medical interventions.
Croatia	Guidelines apply to medical devices but there are no specific regulations
Denmark	Not mentioned
England	Separate document with recommendations for the evaluation of MDs: Medical Technologies Evaluation Programme Methods Guide, 2017
Europe (EUetHTA advice, not binding)	CCA recommended for MD
Estonia, Latvia, Lithuania	Not mentioned
Finland	Not mentioned (there is an additional guideline only available in Finnish, which might include additional information)
France	Haute Autorite´ de Sante´. Medical device assessment in France: guidebook. Paris: Haute Autorite´ de Sante´; 2009.
Germany	Not mentioned
Hungary	Are given as an example of the necessity of indirect comparisons but not specifically mentioned beyond that
Ireland	Guidelines apply to medical devices but there are no specific regulations

Netherlands	> No lifelong time horizon<br>> Diversity of MDs and user experience should be considered.<br>> Greater ease of use can be considered as value<br>> Differentiation between diagnostic and therapeutic MDs<br>> Technical effectiveness as an additional dimension<br>> CEA is recommended<br>> Discrete-choice experiment (DCE) and multi-criteria decision analysis (MCDA) to assess the full spectrum of value<br>> Utilization degree influences the fixed price per unit and thus the cost<br>> Distinction between collective devices and individual devices
Norway	Not mentioned (however, there is an additional guideline only available in Norwegian, which might address MDs)
Poland	Guidelines apply to medical devices but there are no specific regulations
Portugal	Not mentioned
Russia	Not mentioned
Scotland	Not mentioned
Spain	Not mentioned
Sweden	Economic evaluation of medical devices<br>Final report (2015)

4 Key issues extracted from the literature

Supplementary Table 8: Shortcomings and recommendations for improvement in the current HTA methodology for medical devices taken from a systematic review of available literature.

Publication	Issues raised	Summary of key points	Conflict of interest
Ginsburg 2005[25]<br><br>Original research	-Organizational impact	-Consider usability in the HTA process as it can be a factor to reduce adverse events	No CoI stated
Steuten 2008 [26]<br><br>Review	-Incremental innovation<br>-Low evidence<br>-Transferability	-A decision-analytic approach, where relative clinical efficacy, resource utilization and unit costs for the target country are substituted in the analysis, is the recommended way of transferring economic-evaluation results of medical technologies	No CoI stated

		- Additional empirical research is needed to determine the relative impact of different transferability factors	
Ferrusi 2009 [27]<br><br>Review	- Dynamic pricing	- Budget justification must account for the up-front investment needed if advanced MDs are to exert long-term benefit<br>- Risk-sharing frameworks hold great promise for addressing the needs of industry and government alike	No CoI stated, one author is industry based
Taylor 2009 [28]<br><br>Review	- Learning curve<br>- Incremental innovation<br>- Low evidence<br>- Diversity	- Bayesian methods for the analysis of trial data have been proposed as an alternative framework for evaluation<br>- Multilevel, latent-curve, and time-series models could be used to explore potential learning effects<br>- 'Coverage with evidence' or 'only in research' policies, is an important output of HTA appraisals of MDs to incentivize data collection after market entry	Main author was a paid consultant of a MD company and member of the NICE Appraisal Committee at the time of publication
Trueman 2009 [29]<br><br>Case study as an example for potential general approach	- Low evidence<br>- Transferability	- Evaluates HTA reports of different settings for the same product to investigate feasibility of transferability between settings<br>- There is a common core dataset considered by most of the agencies, which had only limited influence on resulting recommendations<br>- Many HTA agencies sought to generate additional primary research from local settings	No CoI stated
Gelijns 2013 [30]<br><br>Original research	- Learning curve<br>- Incremental innovation<br>- Low evidence<br>- Organizational impact	- Innovative designs for pre-marketing device trials, such as combining nonrandomized and randomized data, adaptive trial designs, and analytical methods to explore temporal and site variations<br>- Build future technological change and learning into sensitivity analyses<br>- Coverage decisions should not be binary "*go/no go*"	No CoI stated
Henshall 2013 [31]	- Dynamic pricing	- There is much common ground between industry, HTA, and coverage bodies, but there are also some important areas of disagreement	Henshall received funding from HTAi for this paper and consultancy fees

Forum discussion protocol		- Central disagreement on whether HTA and coverage bodies acknowledge all relevant elements of value in their assessments	from several medical companies for advisory-board chairing.
Kirisits 2013 [32]<br><br>Review	- Low evidence<br>- Learning curve<br>- Organizational impact<br>- Incremental innovation<br>- Diversity<br>- Dynamic pricing<br>- Transferability	- Current regulatory framework for MDs limits the availability of high-quality evidence at the time of reimbursement decisions<br>- Issues such as reusability, procedural integration and temporal changes in determinants of MDs challenge the calculation, validity and the ability to generalize the cost effectiveness of MDs	No CoI stated
Kingkaew 2014 [33]<br><br>Review	- Learning curve<br>- Incremental innovation<br>- Dynamic pricing<br>- Low evidence<br>- Diversity<br>- Organizational impact	- Costs and outcomes resulting from treatments of the screened or diagnosed diseases should be considered<br>- Where the cost-effectiveness of the MD under consideration depends on other factors such as distribution or usage, a feasibility analysis for such MDs in the context of analysis is also recommended<br>- Where the value of the MD under consideration depends on economies-of-scale, both fixed and variable costs should be obtained in order to calculate the break-even point	No CoI stated
Husereau 2014 [34]<br><br>Forum discussion protocol	- Low evidence	- Replace single-decision points with periodic or staged assessment and re-assessment using an evolving evidence base<br>- Adaptive approaches would have to be developed differently for pharmaceuticals and MDs	Authors associated with HTAi
Rosina 2014 [35]<br><br>Original research	- Learning curve<br>- Incremental innovation<br>- Dynamic pricing<br>- Low evidence<br>- Diversity	- Evaluating the effect of MDs within CEAs using multiple-criteria decision analysis (MCDA) is suggested	No CoI stated

Ferguson 2014 [36]<br><br>Editorial	-Learning curve<br>-Incremental innovation<br>-Dynamic pricing<br>-Low evidence<br>-Diversity<br>-Organizational impact	- It makes sense to perform HTAs intermittently or when an impactful change in safety or effectiveness is suspected<br>- Budgetary time frames should be long enough to account for potentially high, up-front costs with long-term benefits of some MDs	The author is employed by Boston Scientific
Ciani 2015 [37]<br><br>Review	-Low evidence<br>-Learning curve<br>-Incremental innovation<br>-Dynamic pricing	- Overview of organization and practices of non-EU HTA agencies in relation to MDs<br>- Raises the question of whether the differences between medical technologies are substantial enough to require a fundamentally different methodological approach to their HTA<br>- Existing primary and secondary scientific methods may provide a sufficient, methodological basis for the HTA of MDs	No CoI stated
Craig 2015 [38]<br><br>Review	-Learning curve<br>-Incremental innovation<br>-Dynamic pricing<br>-Low evidence<br>-Diversity<br>-Organizational impact	- Use of early models or ‘coverage with evidence development’ initiatives to fund studies of the device in a clinical setting<br>- Evaluation of the Bayesian methods to handle uncertainties and define further research priorities<br>- Healthcare purchasers should be encouraged to increase their knowledge of economic evaluation methods through training activities	No CoI stated
Henschke 2015 [39]<br><br>Original research	-Learning curve<br>-Low evidence<br>-Diversity<br>-Organizational impact	- Implement a category system by which MDs can be stratified<br>- Appropriate HTA methodologies can be defined for each category to assess the evidence needed for a new MD after it has been assigned to one of the categories	No CoI stated
Iglesias 2015 [40]<br><br>Review	-Learning curve<br>-Incremental innovation<br>-Dynamic pricing<br>-Low evidence<br>-Diversity<br>-Organizational impact	- Bayesian methods are particularly well suited to address issues associated with a complex and fragmented evidence base<br>- Hierarchical models provide a powerful platform to estimate the clinical effect associated with incremental innovation<br>- Bayesian hierarchical models for clustered data to deal with learning curve effect or to conduct network meta-analysis<br>- Flexible and creative RCT-design methods, discrete-choice experiments, mixed methods for	No CoI stated

		Bayesian expert elicitation and Bayesian statistical methods represent a powerful toolkit to generate robust clinical evidence	
Dervaux 2015 [41]<br>Round-table discussion	-Diversity<br>-Organizational impact<br>-Transferability	- Assessment must integrate process criteria (time, cost, ease of use etc.)<br>- Either as an endpoint of separate studies or an additional dimension to existing study designs	Mostly industry-associated experts
Schnell-Inderst 2015 [42]<br>Review	-Low evidence<br>-Learning curve<br>-Organizational impact<br>-Incremental innovation<br>-Diversity	- Primary research, like RCTs and long-term registries, is a central demand for evaluation of comparative effectiveness of MDs<br>- Consequence of scarce and low-quality evidence is higher uncertainty for the decision maker	No CoI stated
Bisschop 2016 [43]<br>Review	-Low evidence	- RCTs should take place in a multi-centre setup to limit bias, reduce potential conflicts of interest, and increase sample size<br>- Systematic reviews and meta-analyses of RCTs can synthesize a more reliable evidence base<br>- Follow-up through clinical registries should be obligatory for at least 5 years after initial approval	No CoI stated
Roussel 2016 [44]<br>Round table discussion	-Learning curve<br>-Low evidence<br>-Diversity<br>-Organizational impact<br>-Transferability	- Methodology for organizational impact assessment exists but is underused due to a lack of knowledge<br>- Key parameters are perspective and time horizon<br>- Organizational impact must be analysed separately, although it can be closely related to other aspects	No CoI stated
Ciani 2017 [45]<br>Review	-Learning curve<br>-Incremental innovation<br>-Low evidence<br>-Organizational impact	- Harmonize risk categorization<br>- Post-marketing surveillance is particularly important in the case of devices<br>- We would encourage a continuous dialogue among all parties involved to agree on the types of clinical and health-economic evidence needed	No CoI stated
Fuchs 2017 [46]	-Low evidence<br>-Learning curve	- Further regulatory changes at European level required to generate adequate clinical data<br>- Approaches such as 'coverage with evidence' development recommended	2 authors work for G-BA (highest decision-

Original research	-Organizational impact<br>-Incremental innovation<br>-Diversity<br>-Transferability	- More alignment of HTA regulatory processes needed to improve current situation<br>- Methodological discussion and collaboration between different stakeholders indicated	making body of joint self-government of physicians, dentists, hospitals and health insurance funds, Germany)
Lesén 2017[47]<br><br>Original research	-Low evidence<br>-Diversity	- Benefits other than direct clinical effects may have a large impact on the patients' well-being, but a standardized method for measuring these benefits is unavailable<br>- A generic questionnaire for patient-reported assessment of non-clinical benefits provided by a MD was designed as an alternative/complementary method to EQ-5D	Authors are employed by Nordic Health Economics AB (consultancy and research company)
Rothery 2017 [48]<br><br>Review	-Learning curve<br>-Incremental innovation<br>-Dynamic pricing<br>-Low evidence	- Risk sharing is an especially useful concept for MDs as there is generally less evidence available<br>- 'Coverage with evidence development' as a useful tool to allow early-market access of innovative technology and simultaneous incentive to continue evidence generation for manufacturers	No CoI stated
Tarricone 2017 [49]<br><br>Introduction to a large-scale research project (MedtechHTA)	-Learning curve<br>-Incremental innovation<br>-Dynamic pricing<br>-Low evidence<br>-Organizational impact	- Summary of several publications associated with the MedtechHTA project<br>- Only names the identified problems and their origins<br>- Proposed solutions are detailed in the individual publications	No CoI stated
Tarricone 2017 [50]<br><br>Review	-Learning curve<br>-Incremental innovation<br>-Dynamic pricing<br>-Organizational impact	- To estimate effects of learning curves, it would be important to perform more post-market entry studies to gather experience on its effects for future HTA reports<br>- Iterative Bayesian approach could be used to identify the optimal timing of the adoption of new versions of a technology which is going through an incremental innovation process<br>- Time horizon is critical due to potentially heavy, upfront organizational costs	No CoI stated

		- Perform more post-market studies in order to gather information on the impact of MD-specific issues. This would allow to more reliably assess these for future modelling	
Tarricone 2017 [51]<br><br>Summary of a large-scale research project (MedtechHTA)	- Learning curve<br>- Incremental innovation<br>- Dynamic pricing<br>- Low evidence<br>- Diversity<br>- Organizational impact<br>- Transferability	- Cooperation between regulator and manufacturer, as well as an early initiation of the HTA in the MD development<br>- Focus of new complex devices on specialized centres to reduce learning-curve effects<br>- Bias adjustment for observational data<br>- Determine how the design of registries would need to be adapted to facilitate economic evaluation<br>- Many detail improvements listed as the summary combines findings from multiple studies	No CoI stated
Varabyova 2017 [52]<br><br>Review & original research	- Learning curve	- To estimate effects of learning, additional data should be collected during RCTs (e.g. operator's and hospital's experience)<br>- Bayesian approach with informative priors is suggested to incorporate learning effects into economic analyses<br>- Framework for estimating intervention-specific learning curves in HTAs is provided	No CoI stated
Bogavac-Stanojević 2018 [53]<br><br>Original research	- Low evidence	- No universal guidance for outcome measurements, cost calculation, performance requirements, use of certain types of economic studies, and economic thresholds for MDs<br>- Assessment of cost-effectiveness is primarily of use to the policymaker and the purchaser, while healthcare provider needs to adopt technology in order to satisfy a recognized unmet need	No CoI stated
Polisena 2018[54]<br><br>Delphi survey	- Learning curve<br>- Incremental innovation<br>- Dynamic pricing<br>- Low evidence<br>- Diversity<br>- Organizational Impact	- A review of existing MD-HTA guidelines was conducted and gaps identified<br>- International experts were questioned on their take on these gaps and their opinion on suggested solutions<br>- Agreement across experts was high<br>- Suggestions are presented in a comprehensive table	No CoI stated

5 Acronyms used in the supplementary tables

**15D**	15 Question quality of life questionnaire
**BIA**	Budget-impact analysis
**CBA**	Cost-beneifit analysis
**CCA**	Cost-consequence analysis
**CEA**	Cost-effectiveness analysis
**CMA**	Cost-minimization analysis
**CoI**	Conflict of interest
**CUA**	Cost-utility analysis
**CVA**	Cost-value analysis
**EQ-5D**	EuroQoL five dimensions quality of life questionnaire
**HTA**	Health technology assessment
**HUI**	Health-utilities index
**IQWiG**	Institut für Qualität und Wirtschaftlichkeit im Gesundheitswesen
**MD**	Medical device
**NICE**	National Institute for Health and Care Excellence
**NHS**	National Health Service (UK)
**PSS**	Personal Social Services (UK)
**QALY**	Quality-adjusted life year
**QOL**	Quality of life

**RCT**	Randomized controlled trial
**SF-6D**	Short-form six-dimensions

6 Reference list of consulted HTA guidelines and literatures

1. Fröschl ,B., Bornschein ,B., Brunner-Ziegler ,S., Conrads-Frank ,A., Eisenmann ,A., Gartlehner ,G., et al.: Methodenhandbuch für Health Technology Assessment Version 1. Austria; 2012

2. Cleemput I, Neyt M, Van de Sande S, Thiry N. ,B.H.C.K.C.: Belgian guidelines for economic evaluations and budget impact analyses: second edition [Internet]. Belgium; https://kce.fgov.be/sites/default/files/page_documents/KCE_183_economic_evaluations_second_edition_Report3.pdf (retrieved March 12, 2016) 2012

3. Methodological Recommendations for presented documentation for assessment of the efficacy, safety, and pharmacoeconomic parameters of medicinal products applying for inclusion in the Positive Drug List. Bulgaria; p. 1–122015

4. Agency for Quality and Accreditation in Health Care. Department for Development Research and Health Technology Assessment.: The Croatian Guideline for Health Technology Assessment Process and Reporting. Croatia; p. 1–412011

5. Kristensen ,F.B., Sigmund ,H.: Health technology assessment handbook Copenhagen: Danish Centre for Health Technology Assessment, National Board of Health [Internet]. Health Technology Assessment. Denmark; http://www.sst.dk/publ/Publ2008/MTV/Metode/HTA_Handbook_net_final.pdf 2007

6. NICE: Guide to the Methods of technology appraisal 2013 (PMG9). Process anhttps://www.nice.org.uk/article/pmg9/resources/non-guidance-guide-to-the-methods-of-technology-appraisal-2013-pdf (2013)

7. NICE: Medical Technologies Evaluation Programme [Internet]. England; http://www.nice.org.uk/aboutnice/whatwedo/aboutmedicaltechnologies/medicaltechnologiesprogramme.jsp 2011

8. Behmane ,D., Lambot ,K., Irs ,A., Steikunas ,N.: Baltic Guideline for Economic Evaluation of Pharmaceuticals (Pharmacoeconomic Analysis). Baltic; p. 1–62002

9. Läkemedelsprisnämnden: APPLICATION INSTRUCTIONS HEALTH ECONOMIC EVALUATION PREPARING. Finland; 2015

10. Haute Autorité de Santé: Choices in Methods for Economic Evaluation. France; 2012

11. HAS (Haute Autorité de Santé): Medical device assessment in France. (December)(2009)

12. Institut für Qualität und Wirtschaftlichkeit im Gesundheitswesen: Allgemeine Methoden

Version 5.0. Germany; 2017

13. National Institute of Pharmacy and Nutrition: PROFESSIONAL HEALTHCARE GUIDELINE ON THE METHODOLOGY OF HEALTH TECHNOLOGY ASSESSMENT. Hungary; 2017

14. Health Information and Quality Authority: Guidelines for the Economic Evaluation of Health Technologies in Ireland. Health Information and Quality Authority Ireland; 2014

15. Zorginstituut Nederland: Guideline for economic evaluations in healthcare. :1–45 https://english.zorginstituutnederland.nl/publications/reports/2016/06/16/guideline-for-economic-evaluations-in-healthcare (2016)

16. Statens legemiddelverk (Norwegian Medicines Agency): Guidelines on how to conduct pharmacoeconomic analyses. Norway; 2012

17. The Agency for Health Technology Assessment and Tariff System: Health Technology Assessment Guidelines Version 3.0. Poland; 2016

18. Emília Alves , da S., Carlos Gouveia ,P., Sampaio ,C., João António ,P., Drummond ,M., Trindade ,R.: GUIDELINES FOR ECONOMIC DRUG EVALUATION STUDIES. Portugal; p. 1–55

19. Research Center for Clinical and Economic Evaluation and Pharmacoeconomics of the Russian State Medical University: Procedure for clinical and economic evaluation of drug lists that are submitted for reimbursement coverage from public health care budget. Decision-making criteria. Russia; 2010

20. Scottish Medicines Consortium (SCM): Guidance to Manufacturers for Completion of New Product Assessment Form (NPAF). Scotland; 2017

21. López-Bastida ,J., Oliva ,J., Antoñanzas ,F., García-Altés ,A., Gisbert ,R., Mar ,J., et al.: Spanish recommendations on economic evaluation of health technologies. Eur J Heal Econ. 11(5):513–20 (2010)

22. Swedish Agency for Technology Assessment and Assessment of Social Services: Assessment of methods in health care. Sweden; 2017

23. The Pharmaceutical Benefits Board: General guidelines for economic evaluations from the Pharmaceutical Benefits Board. Sweden; 2003

24. The Dental and Pharmaceutical Benefits Agency, Blixt ,M., Södergård ,B., Hiort ,S., Nilsson ,C., Eckard ,N.: Economic evaluation of medical devices [Internet]. Sweden; www.tlv.se 2015

25. Ginsburg ,G.: Human factors engineering: A tool for medical device evaluation in hospital procurement decision-making. J Biomed Inform. 38(3):213–9 (2005)

26. Steuten ,L., Vallejo-Torres ,L., Young ,T., Buxton ,M.: Transferability of economic evaluations of medical technologies: A new technology for orthopedic surgery. Expert Rev

Med Devices. 5(3):329–36 (2008)

27. Ferrusi ,I.L., Ames ,D., Lim ,M.E., Goeree ,R.: Health Technology Assessment From a Canadian Device Industry Perspective. J Am Coll Radiol. 6(5):353–9 (2009)

28. Taylor ,R.S., Iglesias ,C.P.: Assessing the clinical and cost-effectiveness of medical devices and drugs: are they that different? Value Health. 12(4):404–6 http://dx.doi.org/10.1111/j.1524-4733.2008.00476_1.x (2009)

29. Trueman ,P., Hurry ,M., Bending ,M., Hutton ,J.: The feasibility of harmonizing health technology assessments across jurisdictions: A case study of drug eluting stents. Int J Technol Assess Health Care. 25(4):455–62 (2009)

30. Gelijns ,A.C., Russo ,M.J., Hong ,K.N., Brown ,L.D., Ascheim ,D.D., Moskowitz ,A.J.: Dynamics of device innovation: Implications for assessing value. Int J Technol Assess Health Care. 29(4):365–73 (2013)

31. Henshall ,C., Schuller ,T.: Health technology assessment, value-based decision making, and innovation. Int J Technol Assess Health Care. 29(4):353–9 (2013)

32. Kirisits ,A., Redekop ,W.K.: The economic evaluation of medical devices: Challenges ahead. Appl Health Econ Health Policy. 11(1):15–26 (2013)

33. Kingkaew ,P., Teerawattananon ,Y.: The economic evaluation of medical devices: challenges. J Med Assoc Thai. 97 Suppl 5(1):S102-7 http://www.ncbi.nlm.nih.gov/pubmed/24964706 (2014)

34. Husereau ,D., Henshall ,C., Jivraj ,J.: Adaptive approaches to licensing, health technology assessment, and introduction of drugs and devices. Int J Technol Assess Health Care. 30(3):241–9 (2014)

35. Rosina ,J., Rogalewicz ,V., Ivlev ,I., Juřičková ,I., Donin ,G., Jantosová ,N., et al.: Health technology assessment for medical devices. Lek a Tech. 44(3):23–36 http://www.scopus.com/inward/record.url?eid=2-s2.0-84929176581&partnerID=tZOtx3y1 (2014)

36. Ferguson ,M.: Medical devices are different to pharmaceuticals in the Health Technology Assessment process. J Comp Eff Res. 3(3):217–9 http://www.ncbi.nlm.nih.gov/pubmed/24969145 (2014)

37. Ciani ,O., Wilcher ,B., Blankart ,C.R., Hatz ,M., Rupel ,V.P., Erker ,R.S., et al.: Health Technology Assessment of Medical Devices: a Survey of Non-European Union Agencies. Int J Technol Assess Health Care. 31(3):154–65 (2015)

38. Craig ,J.A., Carr ,L., Hutton ,J., Glanville ,J., Iglesias ,C.P., Sims ,A.J.: A Review of the Economic Tools for Assessing New Medical Devices. Appl Health Econ Health Policy. 13(1):15–27 (2015)

39. Henschke ,C., Panteli ,D., Perleth ,M., Busse ,R.: Taxonomy of medical devices in the logic of health technology assessment. Int J Technol Assess Health Care. 31(5):324–30 (2016)

40. Iglesias ,C.P.: Does assessing the value for money of therapeutic medical devices require a flexible approach? Expert Rev Pharmacoeconomics Outcomes Res. 15(1):21–32 (2015)

41. Dervaux ,B., Szwarcensztein ,K., Josseran ,A., Barna ,A., Carbonneil ,C., Chevrie ,K., et al.: Evaluation et impact non clinique des dispositifs médicaux. Therapie. 70(1):57–62 http://linkinghub.elsevier.com/retrieve/pii/S004059571631040X (2015)

42. Schnell-Inderst ,P., Mayer ,J., Lauterberg ,J., Hunger ,T., Arvandi ,M., Conrads-Frank ,A., et al.: Health technology assessment of medical devices: What is different? An overview of three European projects. Z Evid Fortbild Qual Gesundhwes. 109(4):309–18 (2015)

43. Bisschop ,A., van Tulder ,M.W.: Market approval processes for new types of spinal devices: challenges and recommendations for improvement. Eur Spine J. 25(9):2993–3003 (2016)

44. Roussel ,C., Carbonneil ,C., Audry ,A., Burtey ,S., Faré ,S., Langevin ,F., et al.: Impact organisationnel: Définition et méthodes d'évaluation pour les dispositifs médicaux. Therapie. 71(1):69–96 (2016)

45. Ciani ,O., Wilcher ,B., van Giessen ,A., Taylor ,R.S.: Linking the Regulatory and Reimbursement Processes for Medical Devices: The Need for Integrated Assessments. Heal Econ (United Kingdom). 26:13–29 (2017)

46. Fuchs ,S., Olberg ,B., Panteli ,D., Perleth ,M., Busse ,R.: HTA of medical devices: Challenges and ideas for the future from a European perspective. Health Policy (New York). 121(3):215–29 http://dx.doi.org/10.1016/j.healthpol.2016.08.010 (2017)

47. Lesén ,E., Björholt ,I., Ingelgård ,A., Olson ,F.J.: Exploration and preferential ranking of patient benefits of medical devices: a new and generic instrument for health economic assessments. Int J Technol Assess Health Care. 33(4):463–71 (2017)

48. Rothery ,C., Claxton ,K., Palmer ,S., Epstein ,D., Tarricone ,R., Sculpher ,M.: Characterising Uncertainty in the Assessment of Medical Devices and Determining Future Research Needs. Health Econ. 26:109–23 http://doi.wiley.com/10.1002/hec.3467 (2017)

49. Tarricone ,R., Torbica ,A., Drummond ,M.: Challenges in the Assessment of Medical Devices: The MedtecHTA Project. Heal Econ (United Kingdom). 26:5–12 (2017)

50. Tarricone ,R., Callea ,G., Ogorevc ,M., Prevolnik Rupel ,V.: Improving the Methods for the Economic Evaluation of Medical Devices. Heal Econ (United Kingdom). 26:70–92 (2017)

51. Tarricone ,R., Torbica ,A., Drummond ,M.: Key Recommendations from the MedtecHTA Project. Heal Econ (United Kingdom). 26:145–52 (2017)

52. Varabyova ,Y., Blankart ,C.R., Schreyögg ,J.: The Role of Learning in Health Technology Assessments: An Empirical Assessment of Endovascular Aneurysm Repairs in German Hospitals. Heal Econ (United Kingdom). 26:93–108 (2017)

53. Bogavac-Stanojevic ,N.: Economic evaluation as a tool in emerging technology assessment. Electron J Int Fed Clin Chem Lab Med. 29(3):196–200 (2018)

54. Polisena ,J., Castaldo ,R., Ciani ,O., Federici ,C., Borsci ,S., Ritrovato ,M., et al.: Health technology assessment methods guidelines for medical devices: How can we address the gaps? The International Federation of Medical and Biological Engineering perspective. Int J Technol Assess Health Care. 34(3):276–89 (2018)
